# Discovery and Characterization of Four Aphelenchid Species from Cultivated Regions of Southern Alberta, Canada

**DOI:** 10.3390/microorganisms12061187

**Published:** 2024-06-12

**Authors:** Maria Munawar, Pablo Castillo, Dmytro P. Yevtushenko

**Affiliations:** 1 Department of Biological Sciences, University of Lethbridge, 4401 University Drive W, Lethbridge, AB T1K 3M4, Canada; maria.munawar@uleth.ca; 2 Institute for Sustainable Agriculture (IAS), Spanish National Research Council (CSIC), Campus de Excelencia Internacional Agrolimentario, ceiA3, Avda. Menendez Pidal s/n, 14004 Cordoba, Spain; p.castillo@csic.es

**Keywords:** *Aphelenchus*, *Aphelenchoides*, fungal feeding, identification, morphology, morphometrics, new species, *Robustodorus*, taxonomy

## Abstract

The nematode family Aphelenchoididiae is considered fungal-feeding, predatory, or root hair feeders. Some members of this family are universally present in agricultural landscapes and are an integral part of soil health and conservation studies. In the present soil nematode biodiversity survey, we detected four species of the genera *Aphelenchus*, *Aphelenchoides*, and *Robustodorus*. Because fungal-feeding nematodes from southern Alberta have not previously been reported, we conducted a detailed morphological and molecular investigation, identifying these species as *Aphelenchus avenae*, *Aphelenchoides limberi*, *Aphelenchoides prairiensis* n. sp. and *Robustodorus paramegadorus* n. sp. The first two species we document as new records from southern Alberta, whereas *A. prairiensis* n. sp. and *R. paramegadorus* n. sp. we describe in detail as new taxa. Briefly, *A. prairiensis* n. sp. is an amphimictic species having 4 lateral lines; hemispherical anteriorly flattened lip region; delicate stylet and swelling-like stylet knobs; excretory pore at the posterior edge of nerve ring. Female tail conical, gradually tapering towards a truncated end with single mucro. Spicule 23.0 (20.0–25.0) µm long having elongated rounded condylus, small, blunt conical rostrum, and lamina that gradually tapers towards the rounded distal end; three pairs of caudal papillae were present on the male tail. *Robustodorus paramegadorus* n. sp., is a parthenogenetic species with 3 lines in the lateral fields; lip region rounded, anteriorly flattened; stylet robust, with knobs rounded to bean-shaped; excretory pore located posterior to nerve ring; reproductive components were quite indiscernible with a short 24.0 (18.0–27.0) µm post-vulval uterine sac; tail conical, ending with pointed to wedge-shaped tip. We performed molecular characterizations for each species and constructed phylogenetic trees to study the phylogenetic relationship of these aphelenchid species. The discovery of *A. prairiensis* n. sp. and *R. paramegadorus* n. sp. indicates that soil nematode diversity is relatively unexplored in southern Alberta. The findings of this study will significantly enhance the identification processes and may contribute towards future soil health and biodiversity efforts.

## 1. Introduction

Fungal- or root hair-feeding nematodes are universally present in agricultural landscapes worldwide, forming an important component of soil ecosystems [1,2,3,4]. Typically, nematodes within a genus tend to use similar food sources [5,6]. However, there are exceptions where some nematode species evolve to become significant parasites of plants. For instance, some members of *Aphelenchoides* Fischer [7] and *Ditylenchus* Filipjev [8] are benign and can be cultured on fungi, whereas several other species of these genera are present on plant quarantine lists [9,10,11]. Therefore, a precise and accurate identification is very important for nematode management, phytosanitary purposes or for the selection of resistant crops to the nematode.In the present study, a correct species delimitation between *Aphelenchoides* spp. was achieved by integrative taxonomical approaches that combined morphological, morphometrical, and molecular data. These methods constitute the basis for the development of posterior species-specific molecular probes, for example, the diagnostic recombinase polymerase amplification (RPA) assays and lateral flow dipsticks (LF), developed by Subbotin [12] for the separation of the strawberry foliar nematode *Aphelenchoides fragariae* from other important foliar pathogens, such as the summer crimp nematode of strawberry *Aphelenchoides besseyi* or the rice white tip nematode *Aphelenchoides oryzae* [12]. Control measures that have been successfully used to manage infestations of aphelenchid species, particularly *Aphelenchoides* spp., on key crops have been recently reviewed by de Souza et al. [13] and included the application of chemical nematicides, cultural practices, crop rotation with nonhost plants, biological control agents, induced resistance (silicon or endophytic microorganisms), and blocking parasitism genes (i.e., using RNA interference (RNAi) or genome editing (typically CRISPR/Cas) to disrupt the targets of the effectors in the plant). Here, we investigated both new and known species of *Aphelenchus* Bastian [14], *Aphelenchoides*, and *Robustodorus* (Goodey [15]) Andrássy [16], recovered from southern Alberta’s arable regions. Among these genera, *Aphelenchoides* is the most species-rich, with 181 known species [17], followed by *Aphelenchus* (12 species), and *Robustodorus* (4 species) [18,19,20,21]. Though these genera are known for their mycetophagy and plant-parasitic tendencies [22,23], none of the member species has previously been reported from southern Alberta. Consequently, when we discovered these species during our soil nematode inventory survey, we performed detailed characterization and identified them to the species level. Our thorough morphological and molecular analyses revealed their identities as *Aphelenchus avenae* Bastian [13], *Aphelenchoides limberi* Steiner [24], and two unique species of *Aphelenchoides* and *Robustodorus*, each displaying distinctive traits. Upon comparison with related species, it appeared that these species have a novel status. Therefore, we formally describe them here as *A. prairiensis* n. sp. and *R. paramegadorus* n. sp. Moreover, *Aphelenchus avenae* and *A. limberi* were documented as new records from southern Alberta. The discovery of *A. prairiensis* n. sp. and *R. paramegadorus* n. sp. indicate that the soil nematode diversity is relatively underexplored in southern Alberta. The findings of this study will significantly enhance identification processes and may contribute towards future soil health and biodiversity research.

## 2. Materials and Methods

### 2.1. Sampling, Nematode Extraction, and Morphological Studies

To investigate the diversity of plant-parasitic nematodes (PPNs) associated with natural vegetation in the marginal areas (uncultivated land on the edges of the fields) of irrigated fields, we conducted a nematode inventory assessment in selected fields of southern Alberta, Canada. Soil samples were collected from the fields using a pointed shovel, and each sample was carefully stored in labelled plastic bags for further analysis. The soil samples were stored in the cold storage facility of the University of Lethbridge (southern Alberta, AB, Canada) until processing. Nematodes were extracted from soil samples using the modified Cobb’s sieving and flotation–centrifugation method [25]. Among other soil nematodes, individual aphelenchid taxa were hand-picked and mounted on slides for observation and preservation. For preliminary examination, freshly isolated *Aphelenchus, Aphelenchoides* and *Robustodorus* taxa were transferred to a drop of distilled water, heat relaxed, and observed under a Zeiss Axioskope 40 microscope (Carl Zeiss, Jena, Germany). Morphological and morphometric characteristics are fundamental for the differential diagnosis of various genera and species. Therefore, we used the following de Man [26] body ratios and other characteristics [27] for the morphometric analysis of the species identified in this study: n, number of specimens on which the measurements are based; a, body length/maximum body diameter; b, body length/distance from anterior end to pharyngo-intestinal junction; b′ body length/distance from anterior end to posterior end of pharyngeal glands; c, body length/tail length; c′, tail length/tail diameter at anus; V, distance from the body anterior end to the vulva as a percentage (%) of the body length; V′, position of vulva from the anterior end expressed as percentage of distance from head to anal aperture. For morphometrical studies, the nematodes were fixed, and permanent slides were prepared as described by De Grisse [28]. Images of each specimen were acquired using a Zeiss Axioskope 40 microscope equipped with a Zeiss Axiocam 208 camera (Carl Zeiss, Jena, Germany). Measurements from the images were performed using ZEN blue 3.1 imaging software (Carl Zeiss).

### 2.2. DNA Extraction, PCR and Sequencing

After microscopic examination, single females of each unidentified species were transferred to a 0.2 mL PCR tube, and the DNA was extracted as described in Maria [29]. Three sets of DNA primers (Integrated DNA Technologies, Coralville, IA, USA) were used to amplify the partial 18S, 28S, and ITS ribosomal RNA (rRNA) genes. The partial 18S rRNA gene sequence was amplified with the 1813F/2646R primer pair [30]. The 28S rRNA gene was amplified using the D2A/D3B primer pair [31], and the ITS gene was amplified using the Vrain2F/Vrain2R primer pair [32]. For the 18S, ITS, and 28S genes, the PCR conditions were as described previously [30,31,32]. Amplified PCR products were resolved by electrophoresis in 1% agarose gels and visualized by staining with GelRed (Biotium, Fremont, CA, USA). The PCR products of appropriate size and sufficiently high quality were sent to Azenta Inc. for purification and sequencing (South Plainfield, NJ, USA). The newly obtained DNA sequences were aligned using the BioEdit sequence alignment tool (BioEdit v. 7.2.5) and compared for similarities with all known nematode species sequences in the GenBank database.

### 2.3. Phylogenetic Studies

The obtained 18S, ITS, and D2–D3 expansion segments of the 28S rRNA gene sequences, and selected aphelenchid species sequences, were used to perform phylogenetic analyses. Outgroup taxa for each dataset were chosen as described in previously published studies [17,20,21,33]. Multiple nucleotide sequence alignments of the different genes were produced using the FFT-NS-2 algorithm of MAFFT v7.450 [34]. Sequence alignments were manually visualized using BioEdit [35]. The sequences were edited manually, and poorly aligned positions were trimmed using a light filtering strategy (up to 20% of alignment positions). Phylogenetic analyses of the sequence datasets were based on Bayesian inference (BI) using MrBayes v3.1.2 [36]. The best-fit model of DNA evolution was obtained using JModelTest v2.1.7 [37] with the Akaike Information Criterion (AIC). The best-fit model, base frequency, proportion of invariable sites, and gamma distribution shape parameters and substitution rates in the AIC were then used in MrBayes for the phylogenetic analyses. The Tamura and Nei model with invariable sites and a gamma-shaped distribution (TrN + I + G) for the 18S, the transversional model with invariable sites and a gamma-shaped distribution (TVM + I + G) for the D2–D3 expansion segments of the 28S rRNA, and the transitional model with a gamma-shaped distribution (TIM1 + G) for the ITS rRNA, were performed with four chains for 4 × 10^6^ generations. A combined analysis of the three ribosomal genes was not undertaken due to some sequences not being available for all species. The Markov chains were sampled at intervals of 100 generations. Two runs were conducted for each analysis. After discarding burn-in samples of 30% and evaluating convergence, the remaining samples were retained for in-depth analyses. The topologies were used to generate a 50% majority-rule consensus tree. Posterior probabilities (PP) were given on appropriate clades. Trees from all analyses were visualized using FigTree software v1.42 [38].

## 3. Results

### 3.1. Description of Aphelenchoides prairiensis *n. sp.*

**Figure 1:**
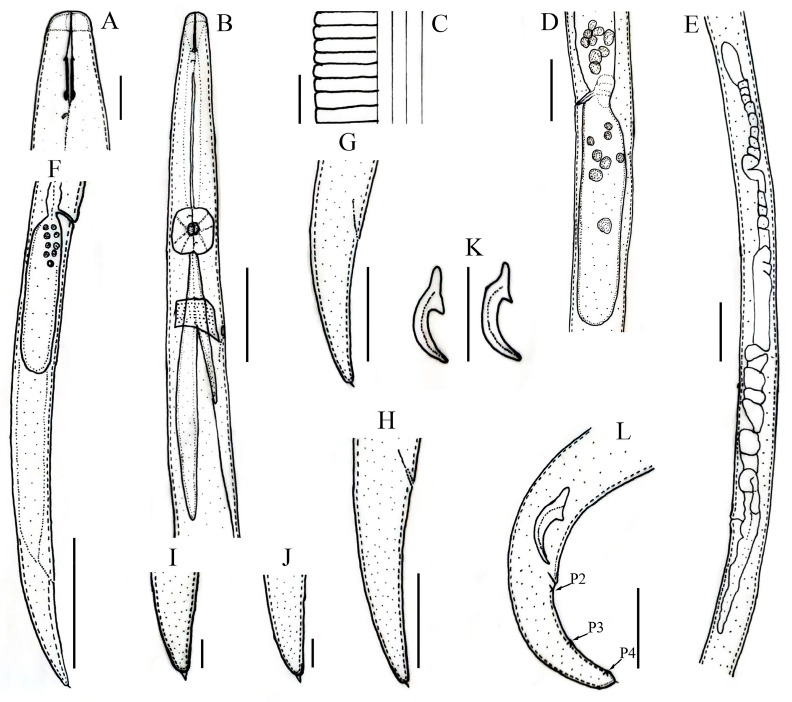
Line drawings of *Aphelenchoides prairiensis* n. sp. male and female. (**A**) Anterior region; (**B**) pharyngeal region; (**C**) lateral field lines; (**D**) vulval region; (**E**) female gonad; (**F**) female posterior body region; (**G**,**H**) female tail regions; (**I**,**J**) female tail tips; (**K**) spicules; (**L**) male tail, arrows pointing towards P2, P3, P4, number of caudal papillae. Vertical lines are scale bars: (**A**,**C**,**I**,**J**) 5 μm; (**F**) 50 μm; (**B**,**D**,**E**,**G**,**H**,**K,L**) 20 μm.

**Figure 2:**
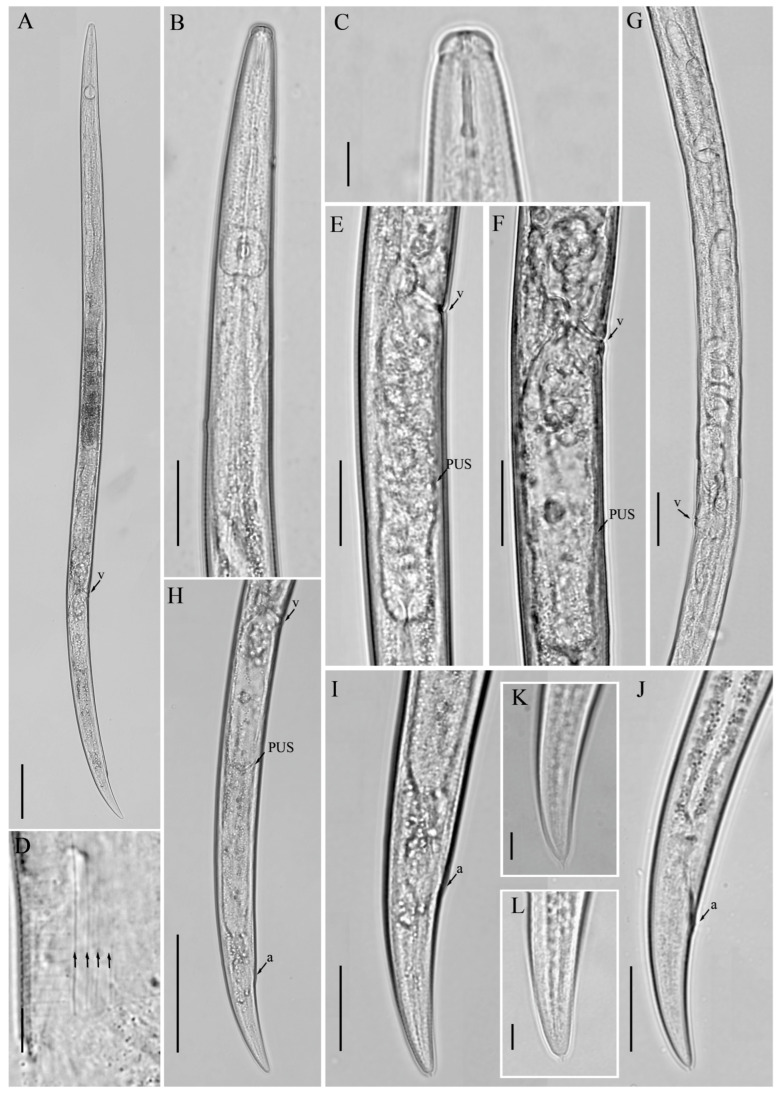
Light micrographs of *Aphelenchoides prairiensis* n. sp. female. (**A**) Entire body; (**B**) pharyngeal region; (**C**) anterior region; (**D**) lateral field lines, arrows indicating the number of lines; (**E**,**F**) vulval region; (**G**) gonad; (**H**) posterior body region; (**I**–**L**) tail regions. Scale bars: (**A**) 50 μm; (**B**,**E**–**I,J**) 20 μm; (**C**,**D**,**K**,**L**) 5 μm. Arrows: (a) anus; (PUS) post-vulval uterine sac; (v) vulva.

*Female:* Body slender, cylindrical, straight to slightly arcuate ventrally with fine annulation. Lateral fields with four incisures. Lip region hemispherical, anteriorly flattened, slightly offset, labial framework lightly sclerotized. Stylet delicate, anterior part of stylet comprising 45–48% of total stylet length. Stylet knobs not well developed, rounded, more like swellings. Median bulb squarish to rounded, valve plates bean-shaped, situated centrally. Pharyngo–intestinal junction less than one stylet length posterior to median bulb. Pharyngeal glands extending dorsally and subdorsally over intestine. Nerve ring located about one body diam. posterior to median bulb. Excretory pore at the posterior edge of nerve ring. In mature females, ovary extending to pharyngeal glands, oocytes in one or two rows, spermatheca elongate oval, with sperm, crustaformeria indistinct, vagina anteriorly directed, vulva simple without any vulval flaps or protruding lips. Post-vulval uterine sac (PUS) conspicuous, large, tubular, occupying the entire body width, empty or containing sperm. Anus distinct, slit-like. Tail conical, gradually tapering towards a truncated end with a single mucro (Figure 1 and Figure 2).

**Figure 3:**
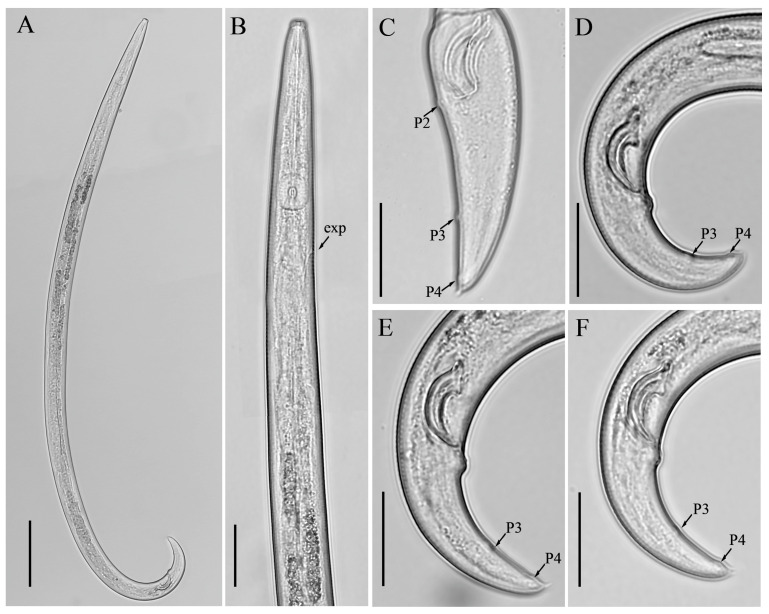
Light micrographs of *Aphelenchoides prairiensis* n. sp. male. (**A**) Entire body; (**B**) pharyngeal region; (**C**–**F**) tail regions. Scale bars: (**A**) 50 μm; (**B**–**F**) 20 μm. Arrows: (exp) excretory pore; (P2, P3, P4) number of caudal papillae.

*Male:* Less frequent than females (proportion 1:5). Body cylindrical attains J-shape after heat relaxed. Anterior end body morphology is similar to that of females. Reproductive system monorchic. Testis outstretched with spermatocytes arranged in single to two rows. Spicules ventrally arcuate, condylus elongated rounded, rostrum small, blunt conical, capitulum with slight to no depression, lamina less wide gradually tapers towards a rounded distal end. Three pairs of caudal papillae (single precloacal P1 absent), the first pair (P2) at cloacal level or slightly posterior to it, the second pair (P3) at about middle of tail, and the third pair (P4), close to the tail tip. Tail similar to that of females, ending with a single mucro (Figure 3).

*Diagnosis and relationship: Aphelenchoides prairiensis* n. sp. is an amphimictic species having four lateral lines; females having slender, cylindrical bodies; hemispherical anteriorly flattened lip region; delicate stylet and swelling-like stylet knobs; excretory pore at the posterior edge of the nerve ring. In mature females, ovary extending to pharyngeal glands; post-vulval uterine sac (PUS) conspicuous, large, tubular, occupying an entire body width, empty or containing sperm. Tail conical, gradually tapering towards a truncated end with a single mucro. Male with moderately long spicule 23.0 (20.0–25.0) µm; condylus elongated rounded, rostrum small, blunt conical, capitulum with slight to no depression, lamina less wide gradually tapers towards the rounded distal end. Caudal papillae are composed of three pairs, P1 absent, P2 at cloacal level, P3 at about the middle of the tail, and P4 close to the tail tip.

*Aphelenchoides* is a species-rich genus; it has more than 180 valid species [17]. For easy identification, the *Aphelenchoides* species were divided into four groups based on tail terminus (group 1: tail end without any outgrowth or mucro; group 2: tail end with one or sometimes two mucronate structures; group 3: star-shaped tail end with four mucronate structures; group 4; tail end with outgrowth other than spine or star). Another stable characteristic in this genus is the number of lateral lines; two to six lateral lines have been reported for *Aphelenchoides* species [39]. By following this scheme, our new species comes close to *A. angusticaudatus* Eroshenko [40], *A. cyrtus* Paesler [41], *A. daubichaensis* Eroshenko [40], and *A. haguei* Maslen [42].

The new species can be distinguished from *A. angusticaudatus* [39] by the presence of males vs. absence; a longer body length of females, 728.0 (564.0–978.0) µm vs. (550–610) µm; a lower b value of females, 9.0 (8.0–10.0) vs. (11–13); a higher c value of females 17.0 (15.0–21.0) vs. (12–18); and a lower c′ value of females, 3.4 (2.8–4.0) vs. 5.1. From *A. cyrtus*, by having an almost straight body habitus of females vs. arc-shaped; swelling type stylet knobs vs. distinctly knobbed; spicule shape (condylus elongated rounded, rostrum small, blunt conical, lamina less wide, gradually tapers towards the rounded distal end vs. (after drawings) condylus and rostrum appear pointed, lamina is wider, distal end appears pointed); the position of the tail mucro (central vs. appears ventrally located); habitat (post-harvest potato field vs. lichens, mushroom amended with flax and straw additives); longer body lengths of females, 728.0 (564.0–978.0) µm vs. (500–570) µm, and males, 720.0 (624.0–859.0) µm vs. (500–510) µm; a higher a value of females, 34.0 (31.0–38.0) vs. (24–28), and males, 33.5 (30.0–36.5) vs. (26–27). From *A. daubichaensis*, by having males vs. the absence; the shape of the median bulb (squarish rounded vs. heart shape); longer PUS length (about 3–5 vulval body width vs. 1 and half vulval body width); shape of tail terminus (truncated vs. peg-like); habitat (post-harvest potato field vs. soil, root system, leaves, and wheat straw); and a longer body length of females, 728.0 (564.0–978.0) µm vs. (467–594) µm. From *A. haguei*, by having simple vs. protrusive vulva lips; the shape of tail mucro (simple vs. multi-papillate); spicule morphology (condylus elongated rounded, rostrum small, blunt conical, lamina less wide, gradually tapers towards the rounded distal end vs. (after drawings) condylus very broad, rostrum small rounded, lamina wider with pointed distal ends); longer body lengths of females, 728.0 (564.0–978.0) µm vs. 624 (560–765) µm, and males, 720.0 (624.0–859.0) µm vs. 604 (525–740) µm; and a higher c value of females, 17.0 (15.0–21.0) vs. 13 (10.6–15.8) (Table 1).

*Type host and locality*: The new species was found in a post-harvest potato field located in Bow Island, southern Alberta, Canada.

*Type material*: Holotype female, 13 paratype females, and 3 males (four slides, numbers UL-DY3-01 to UL-DY3-04) were deposited in the nematode collection of the University of Lethbridge, AB, Canada. Four paratype females and two paratype males were deposited in the nematode collection of the Institute for Sustainable Agriculture, CSIC, Córdoba, Spain.

*Etymology*: The species epithet is derived from the Latin word “*prairiensis*” meaning “of the prairie”. It indicates the origin or habitat of the species, suggesting that it is found in prairie regions of southern Alberta.

### 3.2. Description of Aphelenchoides limberi Steiner [24]

*Female:* Body slender, cylindrical, arc-shaped when heat relaxed. Lateral fields with four incisures. Lip region rounded, anteriorly truncated, slightly offset, labial framework slightly sclerotized. Stylet delicate, anterior part of stylet comprising 48–50% of total stylet length. Stylet knobs weakly developed, rounded, more like swellings. Median bulb rounded, valve plates bean-shaped, situated centrally. Pharyngo–intestinal junction slightly posterior to median bulb. Pharyngeal glands extend dorsally and subdorsally over intestine. Nerve ring located less than one body diam. posterior to median bulb. Excretory pore at the posterior edge of nerve ring. In mature females, ovary reflexed, oocytes in one or two rows, spermatheca and crustaformeria indistinct, vagina anteriorly directed, vulva with protruding lips. Post-vulval uterine sac (PUS) conspicuous, large, tubular, occupying the entire body width. Anus distinct, slit-like. Tail cylindrical, gradually tapering towards a truncated end without any mucro.

*Male:* Not found.

**Figure 4:**
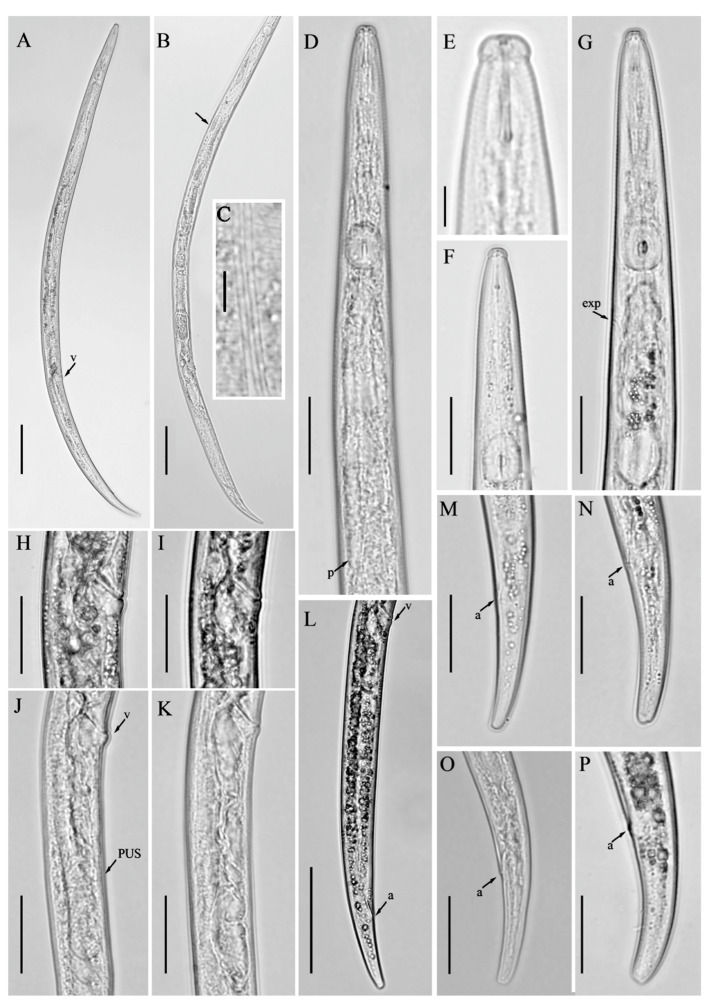
Light micrographs of *Aphelenchoides limberi* Steiner [24], female. (**A**,**B**) Entire bodies, genital track arrowed; (**C**) lateral lines; (**D**,**G**) pharyngeal regions; (**E**,**F**) anterior regions; (**H**–**K**) vulval regions; (**L**) posterior body region; (**M**–**P**) tail regions. Scale bars: (**A**,**B**) 50 μm; (**D**,**F**–**P**) 20 μm; (**C**,**E**) 5 μm. Arrows: (a) anus; (exp) secretory–excretory pore; (p) pharyngeal lobe; (PUS) post-vulval uterine sac; (v) vulva.

*Remarks:* The species was initially described in Juno plant examinations imported from Holland [24]. The species was sporadically mentioned in the literature, with records from Ottawa, Canada [43], Iran [44], and the Czech Republic [45]. However, none of these sources provided photographic evidence or comprehensive morphometric data, except for the latter. Our morphometric data align well with the characteristics observed in both the original description and the population from the Czech Republic. The host associations of *A. limberi* were reported as tulip bulbs (Ottawa, ON, Canada), vineyards (Markazi, Iran), and hop gardens (Senice na Hané, Czech Republic). In our study, we identified *A. limberi* in a post-harvest potato field of southern Alberta and performed detailed morpho-molecular analyses. Consequently, our data can be used in future integrated taxonomical studies (Figure 4; Table 2).

### 3.3. Description of Aphelenchus avenae Bastian [14]

**Figure 5:**
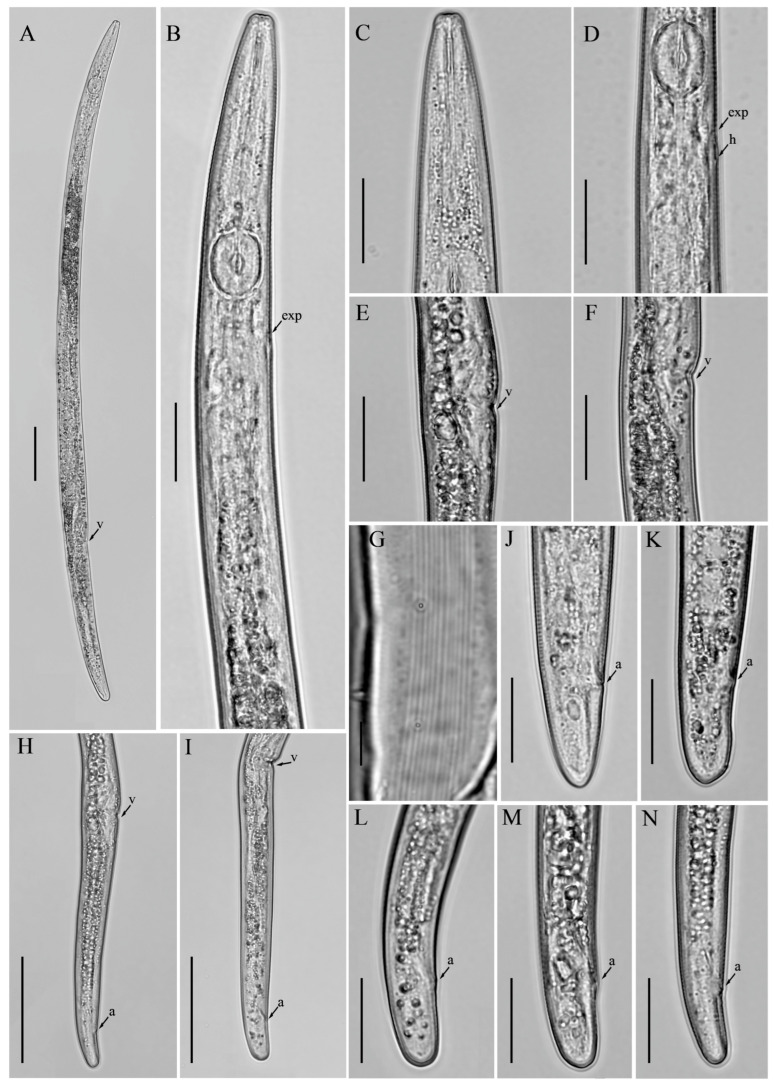
Light micrographs of *Aphelenchus avenae* Bastian [14], female. (**A**) Entire body; (**B**) pharyngeal region; (**C**) anterior region; (**D**) posterior pharyngeal region; (**E**,**F**) vulval regions; (**G**) lateral lines; (**H**,**I**) posterior body regions; (**J**–**N**) tail regions. Scale bars: (**A**,**H**,**I**) 50 μm; (**B**–**F**,**J**–**N**) 20 μm; (**G**) 5 μm. Arrows: (a) anus; (exp) secretory–excretory pore; (h) hemizonid; (v) vulva.

*Female:* Body slender, cylindrical, slightly arched when heat relaxed. Lateral fields with twelve incisures. Lip region squarish rounded, anteriorly flattened, slightly offset. Stylet delicate, without stylet knobs. Median bulb oval rounded, valve plates bean-shaped, situated centrally. Pharyngo–intestinal junction less than one stylet length posterior to median bulb. Pharyngeal glands extend dorsally over intestine. Nerve ring located about one body diam. posterior to median bulb. Excretory pore at the anterior edge of nerve ring. Gonad monodelphic, anteriorly outstretched, oocytes in one or two rows, spermatheca and crustaformeria indistinct, vagina anteriorly directed, vulva lips simple with deep depression. Post-vulval uterine sac (PUS) conspicuous, large, occupying the entire body width. Anus distinct, slit-like. Tail cylindrical, gradually tapering towards a broadly rounded terminus (Figure 5; Table 3).

*Male:* Not found.

*Remarks: Aphelenchus avenae* has global distribution and a wider host range [2]. This species was detected in several of our soil samples collected from cultivated and post-harvest potato fields. However, our focus in this study is to document its presence in southern Alberta. Morphometric or morphological comparisons across all reported populations of *A. avenae* are not within the purview of this research.

*Fungal feeding tests*: The Potato Research Laboratory at the University of Lethbridge is dedicated to studying major plant pathogens associated with potato diseases. Our laboratory maintains a diverse collection of fungal pathogens known to affect potatoes. Here, we selected two major fungal pathogens (*Phytophthora infestans* and *Fusarium sambucinum*) to study nematode reproduction. The rationale for selecting only two fungal pathogens was as follows: (i) the labor-intensive process of nematode extractions and the hand-picking of select *Aphelenchoides* species from soil nematode suspensions; (ii) the limited availability of nematodes; and (iii) this study’s focus, which was solely to provide taxonomic descriptions of these nematode species. For the fungal-feeding test, thirty individuals of *A. limberi* and *A. prairiensis* n. sp. were surface-sterilized using streptomycin sulphate and separately introduced to 14-day-old mycelial mats of the aforementioned fungal pathogens and incubated in the dark for two months, as described by Castillo et al. [10] and Munawar et al. [46] Microscopic observations revealed no evidence of nematode reproduction, indicating that *P. infestans* and *F. sambucinum* are not suitable hosts for *A. limberi* and *A. prairiensis* n. sp.

### 3.4. Description of Robustodorus paramegadorus *n. sp.*

**Figure 6:**
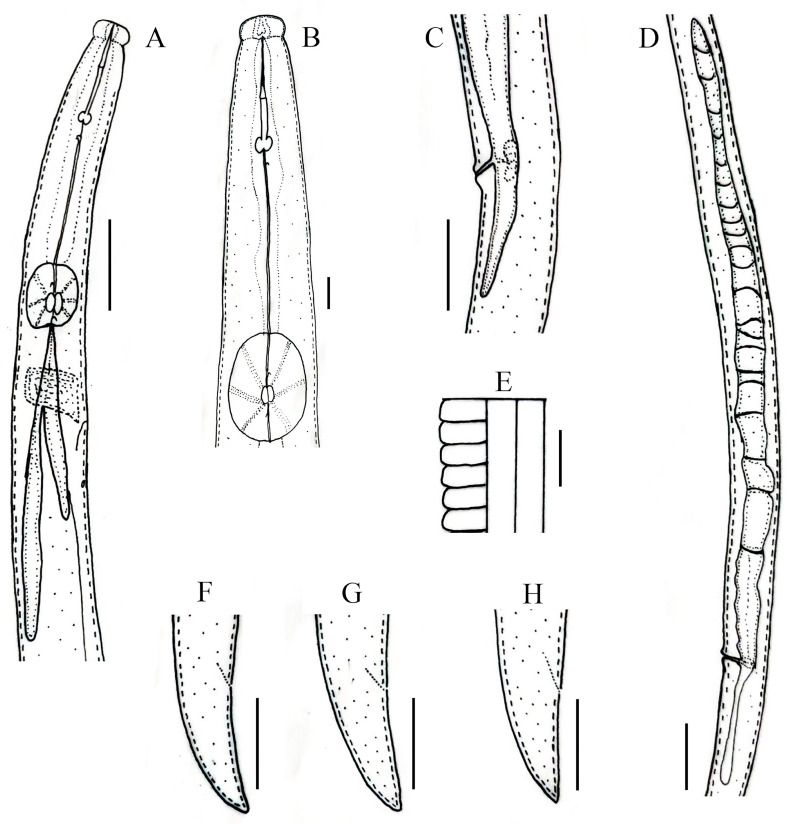
Line drawings of *Robustodorus paramegadorus* n. sp. Female. (**A**) Pharyngeal region; (**B**) anterior region; (**C**) vulval regions; (**D**) gonad; (**E**) lateral lines; (**F**–**H**) tail regions. Scale bars: (**A**,**C**,**D**,**F**–**H**) 20 μm; (**B**,**E**) 5 μm.

**Figure 7:**
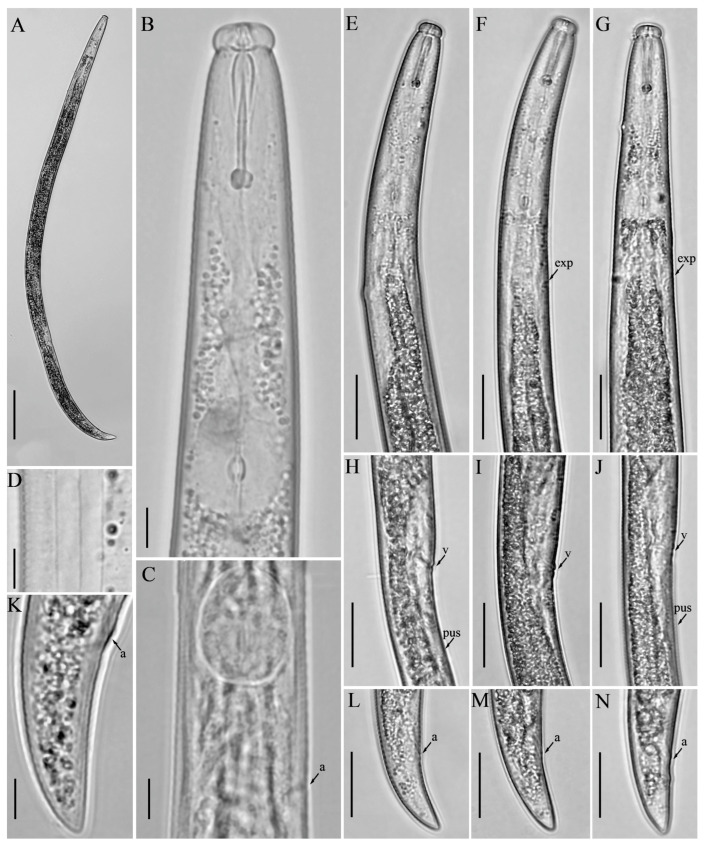
Light micrographs of *Robustodorus paramegadorus* n. sp. female. (**A**) Entire body; (**B**) anterior region; (**C**) median bulb region; (**D**) lateral lines; (**E**–**G**) pharyngeal regions; (**H**–**J**) vulval regions; (**K**–**N**) tail regions. Scale bars: (**A**) 50 μm; (**E**–**J**,**L**–**N**) 20 μm; (**B**–**D,K**) 5 μm. Arrows: (a) anus; (exp) secretory–excretory pore; (PUS) post-vulval uterine sac; (v) vulva.

*Female:* Body cylindrical, slightly arched when heat relaxed. Lateral fields with three incisures. Lip region rounded, anteriorly flattened, with deep constriction. Stylet robust, anterior part of stylet comprising 45–48% of total stylet length. Stylet knobs rounded to bean-shaped. Median bulb squarish to rounded, valve plates ellipsoidal-shaped, situated slightly posteriorly. The gland duct of the dorsal pharyngeal gland opens into the lumen of the median bulb just anterior to the valve; the sub-ventral glands ducts open immediately after the valve. Pharyngo–intestinal junction less than one stylet length posterior to median bulb. Pharyngeal glands extend dorsally and subdorsally over intestine. Nerve ring located 10–12 µm posterior to median bulb. Excretory pore located posterior to nerve ring. Hemizonid one median bulb length behind excretory pore. Reproductive components quite indiscernible, gonad outstretched, not extending to pharyngeal glands, oocytes in one or two rows, spermatheca and crustaformeria indistinct, vagina anteriorly directed, vulva simple without any vulval flaps, both vulval lips slightly protruding. Post-vulval uterine sac (PUS) conspicuous, hollow, tubular, occupying less than half of body width. Anus distinct, slit-like. Tail conical, ending with a pointed to wedge-shaped tip (Figure 6 and Figure 7; Table 4).

*Male:* Not found.

*Diagnosis and relationship:* The new species appears to be parthenogenetic, because males were not found and no sperm was found in indistinct/irregular shaped spermatheca. It has a cylindrical, slightly arched body with three lines in the lateral fields. Lip region rounded, anteriorly flattened, with deep constriction. Stylet robust, with rounded to bean-shaped knobs. Median bulb squarish to rounded, valve plates ellipsoidal-shaped, situated slightly posteriorly. Nerve ring located 10–12 µm posterior to the median bulb. Excretory pore located posterior to nerve ring. Reproductive components are quite indiscernible, vagina anteriorly directed, vulva simple without any vulval flaps, both vulval lips slightly protruding. Post-vulval uterine sac (PUS), short 24.0 (18.0–27.0) µm, conspicuous, hollow, and tubular. Tail conical, ending with a pointed to wedge-shaped tip.

The genus has four nominal species, *R. arachidis* Bos [47], *R. helicus* Heyns [48], *R. megadorus* (Allen [49]) Andrássy [16], and *R. subtenuis* Cobb [50]. *Robustodorus paramegadorus* n. sp. is most similar to *R. megadorus* by Allen [45], and the *R. megadorus*’s Utah population by Ryss et al. [18]. *Robustodorus paramegadorus* n. sp. can be differentiated from *R. megadorus* by the shape of the stylet knobs (rounded to bean-shaped vs. C-shaped); the position of the excretory pore (posterior to nerve ring vs. between median bulb and nerve ring); position of the hemizonid (one median bulb length behind excretory pore vs. two–three annuli posterior to excretory pore); the shape of the median bulb (squarish rounded vs. rounded ovoid); the position of the median bulb valve plates (posteriorly located vs. central); protrusion of the vulval lips (both vs. anterior); tail tip shape (pointed to wedge-shaped vs. rounded); longer body length of females, 555 (466–623) µm vs. 496 (455–546) µm; longer stylet length of females, 19.5 (18–21) µm vs. 18 (17–19) µm; and longer tail length of females, 24 (21–26) µm vs. 19 (17–20) µm.

From *R. arachidis* [20], it can be differentiated by the absence of males vs. the presence; the shape of the lip region (rounded, anteriorly flattened vs. squarish round); the number of lateral lines (three vs. two–four); tail tip shape (pointed to wedge-shaped vs. broadly rounded with or without mucro); the smaller body length of females, 555.0 (466.0–623.0) µm vs. 690 (600–783) µm; the slimmer body of females; a value, 29.0 (26.0–32.0) vs. 38.5 (34.8–40.9), the longer stylet length of females, 19.5 (18–21) µm vs. 10.4 (10.0–10.6) µm; and a shorter PUS length, 24.0 (18.0–27.0) µm vs. 124 (106–144) µm.

From *R. helicus*, by the absence of males vs. the presence; stylet knob shape (rounded to bean-shaped vs. teardrop shape; the position of the excretory pore (posterior to nerve ring vs. between the median bulb and the anterior edge of the nerve ring); tail tip shape (pointed to wedge-shaped vs. rounded); the longer body length of females, 555.0 (466.0–623.0) µm vs. 464 (395–547) µm; the longer stylet length of females, 19.5 (18–21) µm vs. 10.0 (10.0–11.0) µm; a shorter PUS length, 24.0 (18.0–27.0) µm vs. 38 (22–55) µm; and posteriorly located vulva, 72.0 (70.0–76.0) vs. 69.3 (64.4–71.1).

From *R. subtenuis* [20,44], by the absence of males vs. the presence; the shape of the lip region (anteriorly flattened vs. anteriorly rounded); shape of stylet knobs (rounded to bean-shaped vs. swelling); the shape of the median bulb (squarish to rounded vs. oval); vulval lips (protruding vs. non-protruding); tail tip shape (pointed to wedge-shaped vs. hemispherical with mucro); the shorter body length of females, 555.0 (466.0–623.0) µm vs. 822 (694–927) µm; the longer stylet length of females, 19.5 (18–21) µm vs. 11.6 (11.0–13.0) µm; and a shorter PUS length 24.0 (18.0–27.0) µm vs. 115 (80–138) µm.

*Type host and locality:* The newly discovered species was identified in the natural vegetation (goldenrod plant) rhizosphere, growing in the marginal land of the Bow Island potato cultivated area in southern Alberta, Canada.

*Type material:* Holotype female, 14 paratype females (five slides, numbers UL-DY4-01 to UL-DY4-05) and an additional slide containing five females were deposited in the nematode collection of the University of Lethbridge, Alberta, Canada. Four paratype females were deposited in the nematode collection of the Institute for Sustainable Agriculture, CSIC, Córdoba, Spain.

*Etymology*: The species epithet refers to Gr. prep. para, alongside of, resembling; N.L. masc. n. *megadorus*, because of its close resemblance to *Robustodorus megadorus*.

### 3.5. Molecular Profiling and Phylogenetic Studies

In this study, the detected aphelenchid species were molecularly characterized using the partial 18S, D2–D3 expansion segments of 28S, and ITS rRNA genes. The newly obtained sequences were deposited in NCBI under the following accession numbers; 18S: PP718956–PP718960 for *A. prairiensis* n. sp.; PP718961–PP718963 for *A. limberi*; PP718964–PP718965 for *A. avenae*; and PP718966–PP718967 for *R. paramegadorus* n. sp.; the D2–D3 expansion segments of 28S: PP718968–PP718972 for *A. prairiensis* n. sp.; PP718973–PP718976 for *A. limberi*; PP718977–PP718978 for *A. avenae*; and PP718979–PP718980 for *R. paramegadorus* n. sp.; and ITS: PP729623–PP729625 for *A. prairiensis* n. sp.; PP729626–PP729628 for *A. limberi*; PP729629 for *A. avenae*; and PP729630–PP729632 for *R. paramegadorus* n. sp.

**Figure 8:**
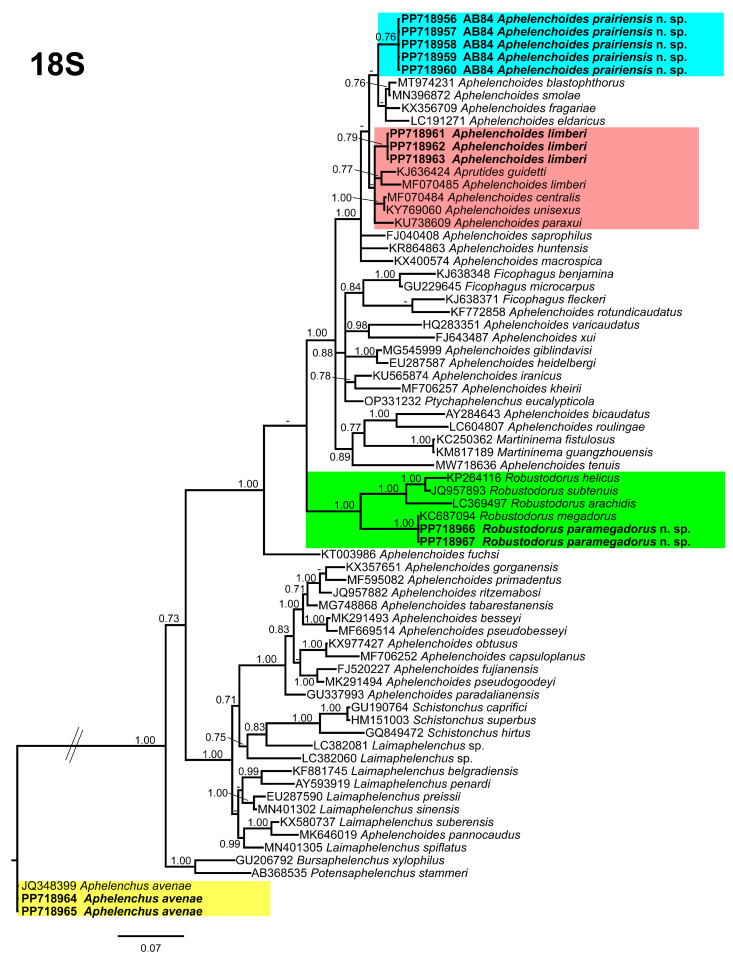
Phylogenetic relationships of the Canadian population of aphelenchid species with the related species. Bayesian 50% majority rule consensus tree as inferred from 18S rRNA sequence alignment under the Tamura and Nei model with invariable sites and a gamma-shaped distribution (TrN + I + G). Posterior probabilities of greater than 0.70 are provided for in the corresponding appropriate clades. The sequences produced in this study are shown in bold, and the colored boxes indicate the clade association of the recovered aphelenchid species.

Phylogenetic trees were constructed for each gene to elucidate the phylogenetic relationships among the detected species and related aphelenchids. The 18S tree (Figure 8), incorporated 71 aphelenchid species sequences, with *Aphelenchus avenae* (JQ348399) serving as the outgroup taxa. Within this tree, *A. prairiensis* n. sp. and *A. limberi* are positioned in the uppermost clade. *Aphelenchoides prairiensis* n. sp. shares a branch with *A. blastopthorus* Franklin [51], *A. fragariae* (Ritzema Bos [52]) Christie [53], *A. eldaricus* Esmaeili, Heydari, Golhasan, and Kanzaki [54], and *A. smolae* Cai, Gu, Wang, Fang, and Li [55]. The sequence identity between *A. prairiensis* n. sp. and the clustered species was 97% (17–21 bp difference), while *A. limberi* formed a subclade with *Aprutides guidetti* Scognamiglio [54], an *A. limberi* population sequence from Iran, *A. centralis* Thorne and Malek [56], *A. unisexus* Jain and Singh [57], and *A. paraxui* Esmaeili, Heydari, Fang, and Li [58]. The sequence identity between *A. limberi* and the clustered species was 97% (15–17 bp difference). *Aphelenchus avenae* clustered with the *A. avenae* outgroup sequence, whereas *R. paramegadorus* n. sp. formed a monophyletic clade with *R. arachidis*, *R. helicus*, *R. megadorus* and *R. subtenuis*. The sequence identity between *R. paramegadorus* n. sp. and the clustered species ranged from 90% to 99% (2–61 bp difference).

**Figure 9:**
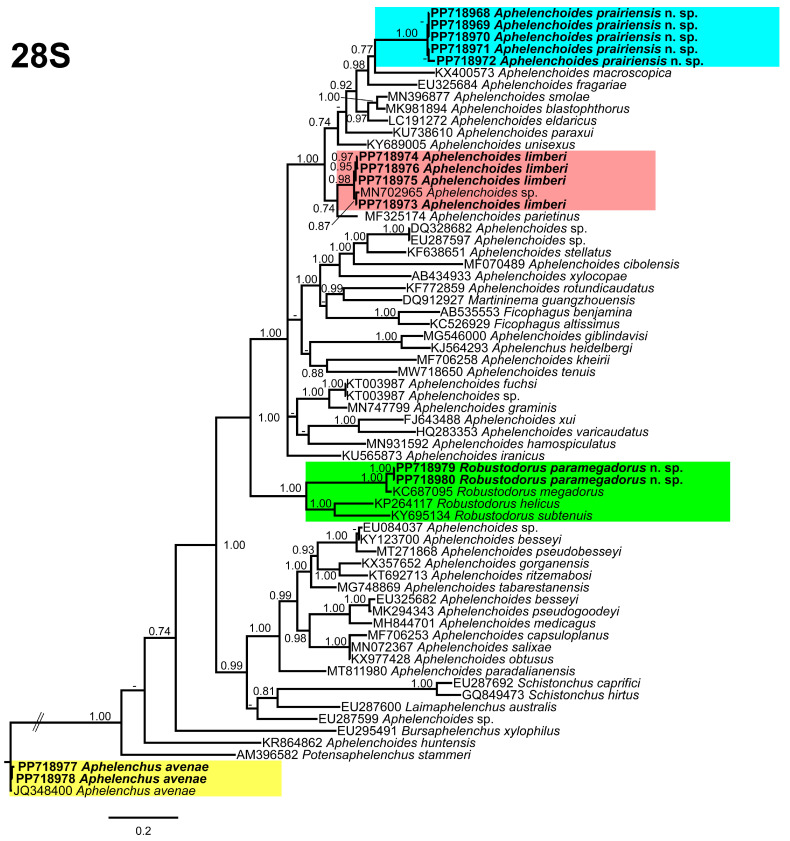
Phylogenetic relationships of the Canadian population of aphelenchid species with the related species. Bayesian 50% majority rule consensus tree as inferred from the D2A–D3B segments of the 28S rRNA sequence alignment under the transversional model with invariable sites and a gamma-shaped distribution (TVM + I + G). Posterior probabilities of greater than 0.70 are provided for in the corresponding appropriate clades. The sequences produced in this study are shown in bold, and the colored boxes indicate the clade association of the recovered aphelenchid species.

The 28S tree (Figure 9), was constructed with 67 aphelenchid species sequences, with *Aphelenchus avenae* (JQ348400) serving as the outgroup taxa. Once again, *A. prairiensis* n. sp. and *A. limberi* were positioned within the top clade of the tree. *Aphelenchoides prairiensis* n. sp. grouped independently with *A. macrospica* Golhasan, Heydari, Esmaeili, and Miraeiz [59]. The sequence identity between *A. prairiensis* n. sp. and *A. macrospica* was 92% (23 bp difference), whereas *A. limberi* was grouped with an unidentified *Aphelenchoides* sp. (MN702965) from Iran and shares a branch with *A. parietinus* (Bastian [14]) Steiner [60]. The sequence identity between *A. limberi* and the clustered species ranged from 96% to 99% (2–10 bp difference). *Aphelenchus avenae* clustered with the *A. avenae* outgroup sequence, whereas *R. paramegadorus* n. sp. formed a monophyletic clade with *R. helicus*, *R. megadorus*, and *R. subtenuis*. The sequence identity between *R. paramegadorus* n. sp. and the clustered species ranged from 77% to 96% (23–147 bp difference).

**Figure 10:**
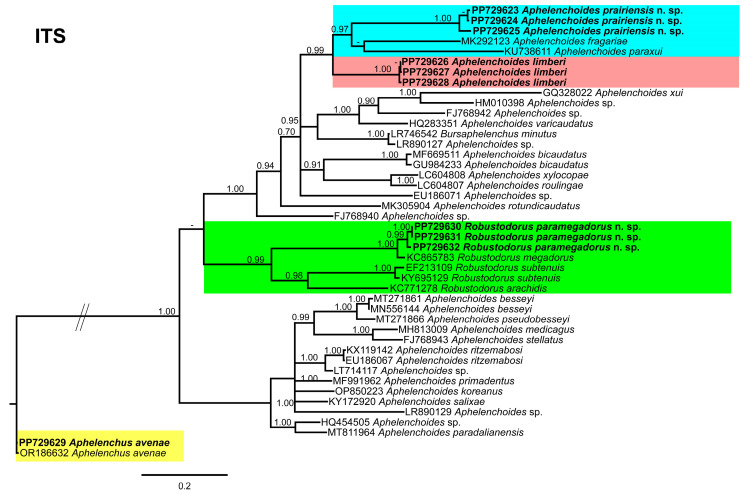
Phylogenetic relationships of the Canadian population of aphelenchid species with the related species. Bayesian 50% majority rule consensus tree as inferred from the ITS rRNA sequence alignment under the transitional model with a gamma-shaped distribution (TIM1 + G). Posterior probabilities of greater than 0.70 are provided for in the corresponding appropriate clades. The sequences produced in this study are shown in bold, and the colored boxes indicate the clade association of the recovered aphelenchid species.

The ITS tree (Figure 10), was constructed using 44 aphelenchid species sequences, with *Aphelenchus avenae* (JQ348400) as the outgroup taxa. Once again, *A. prairiensis* n. sp. and *A. limberi* were positioned within the top clade of the tree. *A. prairiensis* n. sp. grouped with *A. fragariae*, *A. paraxui* and our population of *A. limberi*. The sequence identity between *A. prairiensis* n. sp. and related species ranged from 80% to 88% (24–68 bp difference). *Aphelenchus avenae* clustered with the *A. avenae* outgroup sequence, whereas *R. paramegadorus* n. sp. formed a monophyletic clade with *R. arachidis*, *R. megadorus*, and *R. subtenuis*. The sequence identity between *R. paramegadorus* n. sp. and the clustered species ranged from 79% to 93% (22–63 bp difference).

In our phylogenetic analyses, it is clear that both *A. prairiensis* n. sp. and *R. paramegadorus* n. sp. represent distinct species. Other than our population of *A. limberi*, we obtained a partial 18S sequence of *A. limberi* from the NCBI database. This sequence lacked accompanying measurements and photo-documentation, rendering validation of its true identity unfeasible. Because no other *A. limberi* sequence data are available in the NCBI, consequently, our *A. limberi* population data can be used for future phylogenetic studies.

## 4. Discussion

The soil-dwelling species of *Aphelenchus*, *Aphelenchoides*, and *Robustodorus* have received comparatively less scientific attention than aphelenchids inhabiting foliage or wood. When found in soil, species of these genera are generally categorized as fungal-feeding nematodes [1,61]. However, fungal-feeding activity has not been observed for *Robustodorus*. The genus remained monotypic for a significant duration, but with increased taxonomic studies and molecular characterization, three *Aphelenchoides* species have been reclassified under *Robustodorus* as *R. arachidis*, *R. helicus*, and *R. subtenuis* [18,19,20]. To date, all documented *Robustodorus* species have been discovered in the rhizosphere of various plants, with no reports of fungal feeding or insect association except for *R. helicus*, which was isolated from decaying wood samples and was capable of multiplying on *Botrytis cinerea* cultures [19,20]. Our *R. paramegadorus* n. sp. was discovered in natural vegetation growing on the marginal land adjacent to a potato-cultivated field. The field samples yielded no evidence of the presence of *R. paramegadorus* n. sp. Consequently, we speculate that this new species is primarily associated with natural plants rather than with cultivated crops.

Regarding *Aphelenchus*, the genus has 12 nominal species, with *A. avenae* receiving the most attention due to its global distribution and potential as a biocontrol agent against various fungal species [2]. Currently, there is no evidence indicating that it poses harm to higher plants. Since *A. avenae* is a well-studied species, our objective was solely to report its presence in southern Alberta; we do not speculate on its role in soil ecology or biodiversity. Concerning the genus *Aphelenchoides*, it comprises species that are associated with plants, fungi, and insects [62]. Some species were reported in the oviducts or elytra of insects [63,64]. The newly discovered and known *Aphelenchoides* species we report here were identified in the soil samples of post-harvest potato fields. We do not anticipate their association with insects; these species may engage in fungal feeding or root hair feeding. It is imperative to note that this study is part of our broader investigation into soil nematode diversity. Among all collected soil samples, this is the first instance of discovering *Robustodorus* sp., (50–70 individuals/500 g soil) from any of our soil samples, whereas *A. limberi* (30–40 individuals/500 g soil) and *A. prairiensis* n. sp., (45–55 individuals/500 g soil) were also relatively uncommon. However, *A. avenae* (20–50 individuals/500 g soil) was prevalent in the majority of soil samples. Overall, the species documented in this study were found in soil samples, and their fungal-feeding behavior and insect associations remain unknown.

Since many soil samples from agricultural and natural systems may comprise mixed aphelenchid species, applying integrative taxonomical approaches (molecular barcoding of single individuals and morphometric analyses) can accurately identify and separate these species. Nevertheless, further studies are required to define the exact distribution patterns and co-occurrence of the four encountered species with each other and/or with co-detected species. For this reason, additional samplings are needed for optimizing nematode sampling and extraction methods to define distribution patterns of these aphelenchids. In this scenario, the recent review by Abd-Elgawad [65] may help to design the most appropriate sampling programs to decipher the pattern distributions of these nematodes and to hypothesize about their active or indirect dispersal process [65].

Our phylogenetic studies provide robust evidence supporting the distinct status of both *A. prairiensis* n. sp. and *R. paramegadorus* n. sp. Across all phylogenetic trees analyzed, *A. prairiensis* n. sp. consistently grouped with various *Aphelenchoides* species, thereby confirming its taxonomic association within the genus. The clustered species exhibit no significant morphological resemblance to *A. prairiensis* n. sp. Based on sequence similarity we propose *Aphelenchoides* sp. sH1 (MN702965) as a population of *A. limberi*. However, no morphological or morphometrical data are available for this unidentified *Aphelenchoides* sp. Concerning *Robustodorus*, our findings align with previous studies [18,19,20], supporting the monophyletic status of *Robustodorus* species.

## 5. Conclusions

Soil nematodes play crucial roles in decomposition and nutrient cycling [61]. Previous studies in southern Alberta were primarily focused on plant-parasitic species [3,4,11]. Our study expands this knowledge by reporting the presence of nematodes falling within a broader category of fungal or root hair feeders. Here, we have provided the detailed identification of four aphelenchid species, supported by detailed photo documentation. Our findings not only provide data that may enhance soil health assessments, but also describe two new aphelenchid species, suggesting an increase in the known soil nematode diversity of southern Alberta. Further research is required to elucidate the exact food sources, dispersal modes, and distribution patterns of these nematodes for a comprehensive understanding of their biodiversity and ecological roles.

## Figures and Tables

**Table 1:** Morphometrics of *Aphelenchoides prairiensis* n. sp. All measurements are in μm and in the form: mean ± standard deviation (range).

	Holotype	Paratype	
Characteristics/Ratios	Female		Male
n	–	17	5
L	753	728.0 ± 133.0 (564.0–978.0)	720.0 ± 95.5 (624.0–859.0)
a	37.7	34.0 ± 1.7 (31.0–38.0)	33.5 ± 2.3 (30.0–36.5)
b	9.7	9.0 ± 0.7 (8.0–10.0)	8.5 ± 0.5 (8.0–9.0)
b′	6.0	5.4 ± 0.5 (4.5–6.0)	5.0 ± 0.5 (4.0–5.5)
c	18.4	17.0 ± 1.7 (15.0–21.0)	16.0 ± 1.1 (15.0–17.5)
c′	4.1	3.4 ± 0.3 (2.8–4.0)	3.0 ± 0.2 (2.7–3.2)
V/T	69.3	71.0 ± 1.6 (68.0–75.0)	57.0 ± 3.5 (55.0–60.0)
V′	94.6	94.0 ± 0.6 (93.0–95.0)	–
Lip height	6.4	6.6 ± 0.4 (6.0–7.5)	3.2 ± 0.3 (2.9–3.7)
Lip width	2.5	2.8 ± 0.3 (2.2–3.5)	6.5 ± 0.4 (6.3–7.2)
Stylet length	12.0	11.5 ± 0.7 (10.0–12.5)	12.0 ± 0.7 (11.0–13.0)
Median bulb length (MBL)	13.0	13.5 ± 1.7 (10.0–16.0)	13.5 ± 1.7 (12.0–16.0)
Median bulb width (MBW)	12.0	12.0 ± 1.4(10.0–14.0)	11.0 ± 1.1 (10.0–13.0)
MBL/MBW	1.1	1.1 ± 0.1(0.9–1.3)	1.2 ± 0.1 (1.1–1.3)
Distance from anterior end to SE pore	79.0	84.0 ± 14.8 (66.0–113.0)	91.0 ± 10.7 (76.0–101.0)
Pharynx length (anterior end to pharyngo-intestinal junction)	78.0	79.0 ± 12.2 (60.0–101.0)	85.0 ± 12.9 (70.0–98.0)
Pharynx length (anterior end to the base of pharyngeal lobe)	126.0	135.0 ± 18.9 (111.0–172.0)	147.0 ± 5.7 (143.0–157.0)
Maximum body width	20.0	21.0 ± 3.5 (17.0–28.0)	21.5 ± 3.4 (17.0–25.0)
Vulva body width (VBW)	21.0	21.0 ± 2.2 (18.0–24.0)	–
Distance b/w vulva to tail terminus	231.0	213.0 ± 37.9 (140.0–275.0)	–
Post-vulval uterine sac (PUS) length	78.0	74.0 ± 12.8 (54.0–100.0)	–
Distance b/w vulva to anus (V-A)	190.0	169.0 ± 33.0 (102.0–223.0)	–
PUS/V-A%	33.8	35.0 ± 3.4 (28.0–41.0)	–
Anal/cloacal body width	10.0	12.5 ± 2.2 (10.0–16.0)	15.5 ± 2.1 (13.0–18.0)
Tail length	41.0	43.0 ± 5.9 (33.0–53.0)	44.0 ± 3.9 (40.0–50.0)
Spicule length curved median line	–	–	23.0 ± 2.0 (20.0–25.0)
Spicule length from condylus to distal tip	–	–	22.0 ± 1.4 (20.0–23.0)

**Table 2:** Morphometrics of female *Aphelenchoides limberi* Steiner [24], reported in the present and previous studies. Our study’s measurements are in μm and in the form: mean ± standard deviation (range), whereas the original and Czech Republic population measurements are in μm and in the form: mean (range).

	This Study	Steiner [24]	Čermák et al. [45]
Characteristics/Ratios	Southern Alberta, Canada	Holland	Czech Republic
n	19	–	24
L	654.0 ± 75.0 (537.0–810.0)	(550–640)	740.0 (639–960)
a	33.0 ± 2.5 (30.0–38.0)	31.8 (30.6–33)	33.1 (29.1–42.4)
b	8.2 ± 0.7 (7.0–10.0)	7.8 (6.5–9.2)	10.4 (9.0–12.0)
b′	4.5 ± 0.4 (4.0–5.4)	–	4.8 (4.3–5.9)
c	16.0 ± 1.2 (13.5–18.0)	–	18.1 (16.5–22.2)
c′	3.7 ± 0.3 (3.0–4.0)	–	3.3 (2.9–3.9)
V	68.5 ± 1.2 (65.0–70.0)	70 (66–72)	69.3 (66.3–71.8)
V′	94.0 ± 0.5 (92.5–94.5)	–	–
Lip height	2.8 ± 0.2 (2.3–3.3)	–	3.1 (2.3–3.6)
Lip width	6.0 ± 0.4 (5.0–6.7)	–	6.7 (6.0–7.2)
Stylet length	11.4 ± 0.9 (10.0–12.5)	–	12.2 (10.6–13.7)
Median bulb length (MBL)	13.5 ± 1.0 (12.0–16.0)	–	–
Median bulb width (MBW)	11.0 ± 1.4 (9.0–14.0)	–	–
MBL/MBW	1.2 ± 0.1 (1.0–1.4)	–	–
Distance from anterior end to SE pore	83.5 ± 4.2 (76.0–89.0)	–	–
Pharynx length (anterior end to pharyngo-intestinal junction)	80.0 ± 3.5 (73.0–85.0)	–	–
Pharynx length (anterior end to the base of pharyngeal lobe)	140.5 ± 8.2 (130.0–157.0)	–	–
Maximum body width	20.0 ± 2.3 (16.0–23.5)	–	22.5 (18.4–32.1)
Vulva body width (VBW)	19.0 ± 2.4 (15.0–23.5)	–	–
Distance b/w vulva to tail terminus	451.0 ± 52.9 (364.0–555.0)	–	–
Post-vulval uterine sac (PUS) length	75.0 ± 8.2 (64.0–91.0)	–	83.2 (63.3–123.7)
Distance b/w vulva to anus (V-A)	161.0 ± 21.8 (124.0–208.0)	–	–
PUS/V-A%	47.0 ± 4.5 (39.0–54.0)	–	–
Anal body width	11.0 ± 1.1 (9.5–14.0)	–	12.3 (10.5–16.2)
Tail length	41.3 ± 3.6 (35.0–47.0)	–	40.9 (34.1–47.1)

**Table 3:** Morphometrics of female *Aphelenchus avenae* Bastian [14], found in this study. Measurements are in μm and in the form: mean ± standard deviation (range).

Characteristics/Ratios	Females
n	15
L	613.0 ± 37.0 (551.0–695.0)
a	35.0 ± 4.0 (31.0–45.0)
b	5.2 ± 0.4 (4.6–6.0)
b′	4.0 ± 0.3 (3.3–4.4)
c	27.0 ± 2.4 (22.0–31.5)
c′	2.0 ± 0.2 (1.5–2.2)
V	76.0 ± 1.5 (73.0–79.0)
V′	96.0 ± 0.3 (95.5–97.0)
Lip height	3.3 ± 0.2 (3.0–3.7)
Lip width	7.5 ± 0.4 (7.2–9.0)
Stylet length	15.0 ± 0.6 (14.0–16.0)
Median bulb length (MBL)	17.5 ± 1.5 (15.0–21.0)
Median bulb width (MBW)	13.5 ± 1.2 (11.0–15.0)
MBL/MBW	1.3 ± 0.1 (1.1–1.5)
Distance from anterior end to SE pore	94.5 ± 3.7 (90.0–104.0)
Anterior end to the base of median bulb	74.0 ± 2.3 (69.0–77.0)
Pharynx length (anterior end to pharyngo–intestinal junction)	118.0 ± 4.0 (112.0–124.0)
Pharynx length (anterior end to the base of pharyngeal lobe)	161.5 ± 8.4 (140.0–170.0)
Maximum body width	17.5 ± 2.0 (15.0–22.0)
Vulva body width (VBW)	16.5 ± 1.5 (15.0–21.0)
Distance b/w vulva to tail terminus	146.0 ± 10.9 (130.0–165.0)
Post-vulval uterine sac (PUS) length	50.5 ± 6.5 (37.0–57.0)
Distance b/w vulva to anus (V-A)	123.0 ± 11.6 (102.0–143.0)
PUS/V-A%	41.0 ± 5.8 (31.5–49.5)
Anal body width (ABW)	12.0 ± 1.3 (10.0–14.0)
Tail length	23.0 ± 1.8 (21.0–28.0)

**Table 4:** Morphometrics of *Robustodorus paramegadorus* n. sp. All measurements are in μm and in the form: mean ± standard deviation (range).

	Female	
Characteristics/Ratios	Holotype	Paratype
n	–	17
L	555.0	555.0 ± 43.3 (466.0–623.0)
a	29.2.0	29.0 ± 1.6 (26.0–32.0)
b	8.5.0	8.0 ± 0.6 (7.0–9.0)
b′	4.7.0	4.5 ± 0.4 (4.0–5.5)
c	21.3.0	23.0 ± 1.7 (20.0–25.5)
c′	2.4.0	2.0 ± 0.1 (2.0–2.4)
V	72.45	72.0 ± 1.4 (70.0–76.0)
V′	95.0	96.0 ± 0.3 (95.0–96.0)
Lip height	7.0	7.5 ± 0.6 (6.0–8.0)
Lip width	3.2	3.0 ± 0.2 (3.0–3.5)
Stylet length	18.0	19.5 ± 0.8 (18.0–21.0)
Median bulb length (MBL)	13.0	13.5 ± 1.0 (12.0–15.0)
Median bulb width (MBW)	11.0	11.0 ± 0.6 (10.0–12.0)
Distance from anterior end to SE pore	75.0	76.0 ± 5.9 (70.0–88.0)
Pharynx (anterior end to pharyngo-intestinal junction)	65.0	69.0 ± 4.2 (63.0–75.0)
Pharynx (anterior end to the base of pharyngeal lobe)	118.0	123.0 ± 10.1 (100.0–137.0)
Maximum body width	19.0	19.0 ± 1.3 (17.0–21.0)
Vulva body width (VBW)	17.0	17.0 ± 1.3 (15.0–19.0)
Distance b/w vulva to tail terminus	153.0	154.0 ± 12.7 (130.0–171.0)
Post-vulval uterine sac (PUS) length	22.0	24.0 ± 3.0 (18.0–27.0)
Distance b/w vulva and anus (V-A)	127.0	130.0 ± 12.0 (108.0–147.0)
PUS/V-A%	17.0	19.0 ± 3.0 (13.0–24.0)
Anal body width (ABW)	11.0	12.0 ± 0.8 (11.0–14.0)
Tail length	26.0	24.0 ± 1.6 (21.0–26.0)

## Data Availability

Data are contained within the article.
